# Odd Chain Fatty Acids; New Insights of the Relationship Between the Gut Microbiota, Dietary Intake, Biosynthesis and Glucose Intolerance

**DOI:** 10.1038/srep44845

**Published:** 2017-03-23

**Authors:** Benjamin J. Jenkins, Kevin Seyssel, Sally Chiu, Pin-Ho Pan, Shih-Yi Lin, Elizabeth Stanley, Zsuzsanna Ament, James A. West, Keith Summerhill, Julian L. Griffin, Walter Vetter, Kaija J. Autio, Kalervo Hiltunen, Stéphane Hazebrouck, Renata Stepankova, Chun-Jung Chen, Maud Alligier, Martine Laville, Mary Moore, Guillaume Kraft, Alan Cherrington, Sarah King, Ronald M. Krauss, Evelyn de Schryver, Paul P. Van Veldhoven, Martin Ronis, Albert Koulman

**Affiliations:** 1 Medical Research Council Human Nutrition Research, Elsie Widdowson Laboratory, Fulbourn Road, Cambridge, CB1 9NL. Affiliated with the University of Cambridge, United Kingdom; 2 Lyon University, INSERM U1060, CarMeN Laboratory and CENS, Claude Bernard University, CRNH Rhône-Alpes, Centre Hospitalier Lyon-Sud, 69310, Pierre-Bénite, France; 3 Children’s Hospital Oakland Research Institute, 5700 Martin Luther King Jr. Way, Oakland, CA 94609, United States of America; 4 Department of Pediatrics, Tungs’ Taichung MetroHarbor Hospital, Taichung 435, Taiwan; 5 Division of Endocrinology and Metabolism/Center for Geriatrics and Gerontology, Taichung Veterans General Hospital, No. 1650, Sec. 4, Taiwan Boulevard, Taichung 407, Taiwan; 6 University of Hohenheim, Institute of Food Chemistry, Garbenstrasse 28, D-70599 Stuttgart, Germany; 7 Faculty of Biochemistry and Molecular Medicine, Biocenter Oulu, University of Oulu, P.O. Box 5400, FI-90014, Finland; 8 UMR CEA-INRA Service de Pharmacologie et d’Immunoanalyse, Laboratoire d’Immuno-Allergie Alimentaire, Université Paris-Saclay, F-91991 Gif-sur-Yvette, France; 9 Laboratory of Gnotobiology, Institute of Microbiology, Czech Academy of Science, Novy Hradek, 549 22, Prague, Czech Republic; 10 Department of Medical Research, Taichung Veterans General Hospital, No. 1650, Sec.4, Taiwan Boulevard, Taichung 407, Taiwan; 11 702 Light Hall, Dept. of Molecular Physiology & Biophysics, Vanderbilt University School of Medicine, Nashville, Tennessee 37232-0615, United States of America; 12 Laboratory of Lipid Biochemistry and Protein Interactions (LIPIT), Campus Gasthuisberg – KU Leuven, Herestraat Box 601, B-3000 Leuven, Belgium; 13 College of Medicine, Department of Pharmacology & Experimental Therapeutics, Louisiana State University Health Sciences Centre 1901 Perdido Str., New Orleans, United States of America; 14 NIHR BRC Core Metabolomics and Lipidomics Laboratory, Level 4, Laboratory Block, Cambridge University Hospitals, University of Cambridge, Cambridge, UK

## Abstract

Recent findings have shown an inverse association between circulating C15:0/C17:0 fatty acids with disease risk, therefore, their origin needs to be determined to understanding their role in these pathologies. Through combinations of both animal and human intervention studies, we comprehensively investigated all possible contributions of these fatty acids from the gut-microbiota, the diet, and novel endogenous biosynthesis. Investigations included an intestinal germ-free study and a C15:0/C17:0 diet dose response study. Endogenous production was assessed through: a stearic acid infusion, phytol supplementation, and a *Hacl1*^*−*/*−*^ mouse model. Two human dietary intervention studies were used to translate the results. Finally, a study comparing baseline C15:0/C17:0 with the prognosis of glucose intolerance. We found that circulating C15:0/C17:0 levels were not influenced by the gut-microbiota. The dose response study showed C15:0 had a linear response, however C17:0 was not directly correlated. The phytol supplementation only decreased C17:0. Stearic acid infusion only increased C17:0. *Hacl1*^*−*/*−*^ only decreased C17:0. The glucose intolerance study showed only C17:0 correlated with prognosis. To summarise, circulating C15:0 and C17:0 are independently derived; C15:0 correlates directly with dietary intake, while C17:0 is substantially biosynthesized, therefore, they are not homologous in the aetiology of metabolic disease. Our findings emphasize the importance of the biosynthesis of C17:0 and recognizing its link with metabolic disease.

Recent findings have shown a negative association between circulating odd chain fatty acids (OC-FAs); pentadecanoic acid (C15:0) and heptadecanoic acid (C17:0), with metabolic disease risk^1^,^2^. Therefore, the determination of factors that affect their levels will be important in understanding the aetiology of metabolic disease, and could provide novel mechanistic insight into possible treatment routes.

According to the literature, the origin of C15:0 and C17:0 has long been attributed to the diet^3^,^4^, specifically from ruminant fat as the main contributor in a typical Western diet. This has been explained by the fact that these two OC-FAs are produced by the rumen microbiome^5^ and then incorporated into the fat deposits of the host animal destined for human consumption. All the current evidence for the dietary source of OC-FAs are based on dietary-assessment-correlation studies^1^,^4^,^6^,^7^. However, unambiguous proof of an exclusive dietary association is still lacking and there have been no investigations into the contributions from non-ruminant gut microbiota or from biosynthesis; such as α-oxidation. Interestingly, in different epidemiological studies that showed clear associations of OC-FAs with a reduced risk of metabolic disease^1^,^2^,^8^ there have been considerable differences in disease risk between C15:0 and C17:0, with C17:0 having the strongest inverse association, indicating different affects/effects.

We used a the combination of both different animal and human studies we are able for the first time to comprehensively study the many aspects of circulating OC-FAs.

## Study Design

In each of the studies the appropriate ethical approval was obtained (see Supplementary material; appendix 1–9), where necessary signed informed consent was achieved, and all experimental protocols were approved by a named institutional and/or licensing committee. Additionally, all methods were performed in accordance with the relevant guidelines and regulations.

Several animal models were utilised in this manuscript; including rodent and canine. The rodent models were used to investigate the dietary and biochemical interactions/mechanisms and it has been previously published that a rodent is a suitable animal model for these investigations. The canine models was used due to the sample volume requirements of the study and it has been previously shown that dogs are a suitably comparable model to human glucose intolerance pathology. The animals were maintained under standard laboratory conditions with water *ad libitum* throughout the experiments. Animal body weight, food intake and health were monitored throughout the study. All samples were stored at sub −20 °C until analysed by gas chromatography/direct infusion with mass spectrometry detection (see Supplementary appendix 16 and 17).

### Germ-free study

Four groups of mice were used to asses gut microbiota influences on the host circulating OC-FAs; two groups were kept on a chow diet (n = 8/group) and two groups on a high-fat diet (n = 6–7/group), from each subset there were both conventional or germ-free groups. Blood was collected after 75–84 days and processes into plasma/serum. All experiments were performed with permission 91–493 of the French Veterinary Services and in accordance to the European Community rules of animal care, and in compliance with Directive 86/608/EEC on the protection of animals used for scientific purposes and recommendation 2007/526/EC of the European Commission. Full detail are shown in Supplementary appendix 11 and 12.

### Dose response study

Sprague-Dawley rats (Harlan, Indianapolis, USA) were overfed using one of five experimental diets (n = 6–9/group) at 17% above matched growth as previously described^9^. The fatty acid composition of each diet and the dietary sources of the lipids are shown in Supplementary appendix 13. Blood was collected after 21 days and processed into serum. All experimental procedures were ethically approved by the Institutional Animal Care and Use Committee at the University of Arkansas for Medical Science.

### Phytol supplementation study

C57BL/6 mice (n = 4–5/group) were used in a phytol supplementation investigation^10^. Mice were fed *ad libitum* the rodent diet (Harlan Teklad, USA). The phytol supplemented group received an additional 0·5% (w/w) phytol added to the diet. After 14 days, blood was drawn and separated into serum. All experiments were executed according to accepted criteria for the humane care and experimental use of laboratory animals. All protocols were approved by the Animal Care and Use Committee of the University of Oulu.

### Dairy fat supplementation study

Healthy human subjects with stable body weight and no family history of diabetes (n = 26) were recruited for an overfeeding intervention study conducted by Alligier and colleagues^11^, where samples were analysed and reported in this manuscript. As previously described^11^, the subjects (age 33 ± 1 years, body weight 79.1 ± 1.8 kg and fat mass 19.6 ± 0.8%) followed a 56 day overfeeding intervention, where they ate an additional 760 kcal/day. The additional energy intake was derived from 100 g of cheese, 20 g of butter, and 40 g of almonds. During the study, the subjects maintained their usual eating and physical activity behaviour (monitored by questionnaires and accelerometers over three five-day periods). After an overnight fast at day zero and day 56, blood was drawn and processed into plasma. All the participants gave signed informed consent following the explanation of the experimental protocol. The protocol was approved by the ethics committee of Lyon Sud-Est according to the French ‘Huriet-Serusclat’ law and the Second Declaration of Helsinki (study registration number NCT00905892; 20^th^ May 2009, www.clinicaltrials.gov).

### Controlled diet study

Samples analysed from the publication by Chiu and colleagues^12^. Results from a subset of 64 participants (23 men and 41 women) were used for the present analyses (see Supplementary appendix 14 for participant eligibility and exclusion criteria).

The participants consumed a baseline diet for four weeks, after which they were randomly assigned to one of two experimental diets (high or low dairy-fat diet) for a further four weeks, the macronutrient composition of the baseline and the experimental diets are shown in Supplementary appendix 15. High-, low-, non-fat dairy products and safflower oil were used to vary fatty acid compositions but maintain total fat content. Body weight was measured weekly and, if needed, energy intake was adjusted to achieve stable weight. All diets met the recommended daily intake for vitamins and minerals. Diet compliance was scored through weekly assessments by the dietitian using interviews, menu checklists, and food receipts. Blood was collected at baseline and at the end of the experimental diets after an overnight fast where it was processed into plasma. Signed informed consent was provided and the protocol was reviewed and approved by the institutional review boards of Children’s Hospital & Research Center at Oakland (dba University of California San Francisco Benioff Children’s Hospital Oakland) and the University of California, San Francisco (study registration number NCT01404897; 22^nd^ July 2011, www.clinicaltrials.gov).

### Stearic acid (C18:0) infusion study

As described previously^13^, Sprague-Dawley rats (n = 11/group) received a C18:0 (250 or 1000 nmol/kg/day) intraperitoneal infusion lasting five weeks, control animals received saline only. Blood was collected after 35 days and processed into serum. The animal study was approved by the Animal Care and Use Committee of Taichung Veterans General Hospital, Taiwan.

### 2-hydroxyacyl-CoA lyase 1 (Hacl1) study

*Hacl1*^*+/−*^ Swiss Webster mice were mated to obtain *Hacl1* deficient mice, as described previously^14^. Wildtype littermates were used as the control group (n = 5–7/group). Mice were fed a regular chow diet. After 14 weeks blood was collected and processed into plasma. All animal experiments were approved by the Institutional Animal Ethical Committee of KU Leuven.

### Glucose intolerance study

Healthy adult mongrel dogs (~9 months of age, starting weight is 20.5 ± 2.9 kg, n = 5) were fed a meat based 52% high fat diet (TestDiet, Indiana, United States of America) for four to eight weeks^15^. Before and after the high fat diet intervention a 180 minute oral glucose tolerance test (OGTT) (0.9 g/kg body weight of glucose polymer administered) was carried out following a 24 hour fast; during each OGTT plasma samples were collected ten minutes before the glucose administration for lipid analysis. The baseline plasma samples were used for fatty acid analysis. The protocol was approved by the Vanderbilt University Institutional Animal Care and Use Committee, and the animals were housed and cared for according to Association for Assessment and Accreditation of Laboratory Animal Care guidelines.

### Statistical Analysis

The statistical approaches utilised were appropriate for specific experimental designs; a paired t-test was used to asses significance/insignificance of an observational change that can be paired within a population, such as between an observation before and after an intervention within the same population (a paired t-test was used in the dairy fat supplementation study; see Table 1). A homoscedastic t-test was used between two unrelated equal variance groups to identify any significance/insignificance between them two groups (a homoscedastic t-test was used in the following studies: phytol supplementation study, controlled diet study, stearic acid (C18:0) infusion study, and the 2-hydroxyacyl-CoA lyase 1 (Hacl1) study. For both the paired and homoscedastic t-tests a value of p ≤ 0.05 was considered significant). A goodness of fit of a trendline expressed as R squared (R^2^) that is greater than 0.75 was considered a strong fit, an R^2^ greater than 0.5 was considered moderate. The area under the curve for the glucose intolerance was calculated via the trapezoidal rule, total area under the curve is the sum of all individually calculated trapezoid areas; individual trapezoid area equals (((y value two + y value one)/2)*(x value two − x value one)).

## Results

### Dose response study

Five groups of rats were subjected to isocaloric high-fat diets, where the composition of the fat in each diet had equal increments of beef tallow from 0% to 11·7% (see Supplementary appendix 13). To validate these being legitimate biomarkers of ruminant fat intake, we compared C15:0 and C17:0 compositions between the diets and the serum levels.

**Figure 1:**
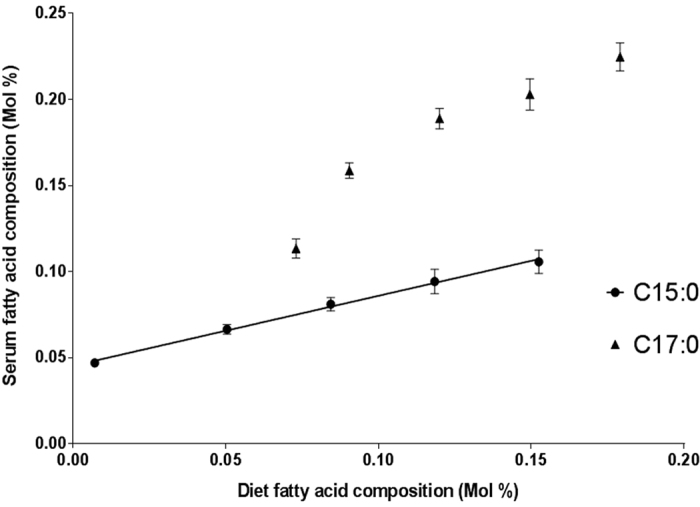
Comparison between the diet composition and the serum composition (Mol%) of pentadecanoic acid (C15:0) and heptadecanoic acid (C17:0) in a dose response study in rats where five groups were subjected to isocaloric high-fat diets with increasing ruminant fat content from 0% to 11·7%. The serum samples were analysed by gas chromatography with mass spectrometry detection. C15:0 levels between diet and blood serum composition is highly correlated, with an R^2^ = 0·997. Error bars represent ± standard error of the mean. (n = 6–9/group).

Increases in C15:0 in the diet linearly correlated (R^2^ = ~1) with serum levels (see Fig. 1). Although C17:0 levels increased, there was not a strong linear response suggesting other influencing factors which do not affect C15:0. Additionally, the smallest difference between C17:0 in each diet is between groups one and two, where there is actually the largest difference in C17:0 levels detected in the serum.

This divergence between these OC-FAs is supported in published literature^4^,^16^,^17^. It has been proposed that C17:0 can also be biosynthesised by α-oxidation^18^; where phytanic acid is the primary target substrate (derived from diet or phytol metabolism^19^). If C17:0 is biosynthesised through α-oxidation, then any increase in the target substrate; phytanic acid, could change the *in vivo* C17:0 levels by increasing or inhibiting the rate of C17:0 biosynthesis^20^. (Phytol and phytanic acid dietary composition shown in Supplementary appendix 19).

### Phytol supplementation study

To directly investigate upregulation or inhibition of C18:0 α-oxidation, we conducted a phytol supplementation study in mice, which is the target substrate for this oxidation pathway. The fatty acids were compared between the groups to assess any influences of the supplementation on the levels of serum OC-FAs.

The levels of C17:0 decreased in the phytol group compared with the control (control: 0·130 ± 0·005 Mol%, phytol: 0·089 ± 0·004 Mol%, homoscedastic t-test between the control and phytol groups; p < 0·001). There was no significant difference seen between the two groups for C15:0 (control: 0·049 ± 0·002 Mol%, phytol: 0·045 ± 0·002 Mol%, homoscedastic t-test between the control and phytol groups; p = 0·174).

### Dairy fat supplementation study

The fatty acid composition of the dietary supplements are shown in Supplementary appendix 20. The plasma C15:0 and C17:0 fatty acid composition are shown in the table below (see Table 1).

Due to the human diet supplementation, the levels of C15:0 increased ~10%. However, C17:0 did not increase from baseline. Dietary assessment showed that the participants maintained their habitual diet alongside the supplementation and that the overfeeding intervention led to a substantial increase in ruminant fats (see Supplementary appendix 21).

### Controlled diet study

The OC-FAs and the substrates involved in α-oxidation were inferred in the diets: baseline, low and high dairy fat diets (see Supplementary appendix 22). The experimental diets varied from the baseline diet by 82% to 152% for C15:0, 78% to 123% for C17:0, increased in phytol by 42% in both diets and phytanic acid varied by 102% to 226% from the baseline diet.

The participants had plasma collected after the baseline diet and again after the experimental diets, the plasma fatty acid changes due to the experimental diet are shown below (see Table 2).

In the high dairy fat diet there was a ~52% increase in C15:0 which caused a ~22% increase in the plasma C15:0. This directly reflects the expected increase by the diet according to the dose response study (see Fig. 1). The low dairy fat diet had ~18% decrease in C15:0 which in turn caused a ~30% decrease in the participant plasma composition; a larger decrease than expected.

The food products used to vary the C15:0 and C17:0 composition of the experimental diets were selected to assess the biomarker response, whilst reducing any risk of pathology to the participants and maintaining palatability over the four week period. Therefore in the high dairy fat diet, milk and cheese were used rather than ruminant tallow. The selected food products have a slightly higher C15:0 to C17:0 composition resulting in a greater overall intake of C15:0. Even though there was a greater C15:0 intake, there was also a significant increase in dietary C17:0, which represented a greater than normal dietary variation; this should have been sufficient to give a significant increase in C17:0 if it was a true dietary biomarker of intake, however, the circulating C17:0 composition decreased across both the experimental diets. To account for this decrease in serum C17:0 we compared the dietary phytol, phytanic acid, and, C17:0 (see Supplementary appendix 22).

### Stearic acid (C18:0) infusion study

The results above suggest that α-oxidation could be important in the biosynthesis of C17:0 but not for C15:0. To investigate if C18:0 (the precursor of C17:0 from α-oxidation) could increase circulating C17:0 we conducted a controlled intraperitoneal infusion of C18:0 in rats and measured the absolute change in C17:0. While there was no significant change in the levels of C15:0 (control: 3·308 ± 0·310 μmol, 250 nmol/kg/day stearic acid: 4·447 ± 0·466 μmol, homoscedastic t-test p = 0·055), there was a significant increase in C17:0 (control: 5·683 ± 0·542 μmol, 250 nmol/kg/day stearic acid: 8·101 ± 0·651 μmol, homoscedastic t-test p < 0·001).

A higher concentration (1000 nmol/kg/day) of infused C18:0 did not significantly increase C17:0 further (p = 0.810), which suggests that C18:0 does not directly increase the rate of α-oxidation on straight chain fatty acids and that C18:0 is just an untargeted substrate.

### 2-hydroxyacyl-CoA lyase 1 (Hacl1) study

To further investigate OC-FAs biosynthesis via α-oxidation we examined a *Hacl1*^*−*/*−*^ mouse model. This gene is responsible for catalysing a carbon-carbon cleavage reaction, removing formyl-CoA from 2-hydroxy-fatty-acyl-CoA to produce a fatty aldehyde that is shorter in chain length by one carbon unit^21^.

The *Hacl1* gene knockout mouse model caused a reduction in C17:0 by 32·5% from the control group (control: 0·200 ± 0·022 Mol%; *Hacl1*^*−*/*−*^: 0·135 ± 0·008 Mol%, homoscedastic t-test p = 0·017). There was no significant difference in any other fatty acid measured (for C15:0: control: 0·023 ± 0·006 Mol%, *Hacl1*^*−*/*−*^: 0·016 ± 0·001 Mol%, homoscedastic t-test p = 0·176).

### Glucose intolerance study

The relationship between C15:0 and C17:0 with glucose intolerance has been reported through a number of epidemiology studies^1^,^8^. Any variation in circulating C15:0 and C17:0 has been attributed to variations in ruminant fat intake with no consideration of biosynthesis. Therefore, we investigated how these OC-FAs correlate with the development of glucose intolerance during a controlled high fat diet in a canine model; the canine model was selected due to the sample volume requirements of the study and it has been previously shown that dogs are a suitably comparable model to human glucose intolerance pathology.

**Figure 2:**
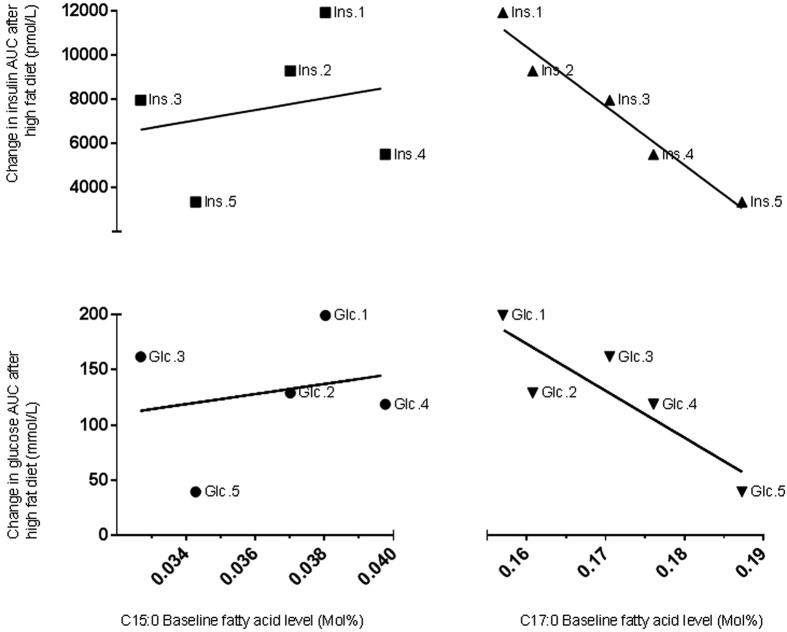
Oral glucose tolerance test (OGTT) performed at the beginning and at the end of a four to eight week high fat diet intervention in dogs. Glucose and insulin were measured from 0 to 180 minutes and the area under the curve (AUC) was calculated using the trapezoidal rule. The graph data points represent the difference between the insulin AUC and the glucose AUC, and from the beginning to the end of the four to eight week high fat diet in relation to the fatty acid levels at baseline (Mol%). Top left: INSULIN (Ins.) - C15:0 R^2^ = 0·053, Top right: INSULIN (Ins.) - C17:0 R^2^ = 0·957. Bottom left: GLUCOSE (Glc.) - C15:0 R^2^ = 0·048, Bottom right: GLUCOSE (Glc.) - C17:0 R^2^ = 0·755. (n = 5).

The glucose and insulin response to an OGTT was determined at baseline and again at the end of a glucose intolerance inducing diet. The difference between the canine baseline OGTT results and the subsequent results were compared to the baseline plasma C15:0 and C17:0 levels (see Fig. 2). Across the intervention, the dogs gained an average of 4.6 ± 1.3 kg.

When comparing other fatty acid levels from baseline with the progression of glucose intolerance in the dogs, only palmitic acid (C16:0) showed a strong positive linear correlation (glucose R^2^ = 0·891, insulin R^2^ = 0·818). This relationship between C16:0 and glucose intolerance has been previously reported in other studies^22^,^23^ and enforces the validity of this study.

## Discussion

This is the first report that combines the results of different animal and human studies to comprehensively investigate the factors that affect the circulating levels of C15:0 and C17:0.

Firstly, we found there were no differences related to the presence or absence of gut microbiota on the mouse circulating OC-FA levels (see Supplementary appendix 18), and hence we can conclude that there was no evidence to suggest that the gut microbiota has an impact on circulating OC-FAs in the mice.

We conducted a five stage ruminant-fat dose response study in rats to assess the relationship between intake of C15:0 and C17:0 with their *in vivo* circulating levels. The diet resulted in a beef-tallow composition varying from 0% to 11·7% by increments of 2·7%, which represent normal ranges of dairy fat intake in typical Western countries. Circulating C15:0 levels directly correlated with intake (see Fig. 1), however, C17:0 does not show the same linear relationship suggesting other influencing factors. There is increasing evidence that C17:0 can be biosynthesised *in vivo*, which could account for its non-dietary origin^24^,^25^.

Several *in vitro* investigations have suggested that C17:0 is produced endogenously both through elongation of propionyl-CoA^26^ and alternatively, through α-oxidation of C18:0^21^,^27^,^28^. However, the results for the C17:0 biosynthesis through propionyl-CoA do not directly explain tissue C17:0 levels in whole organisms, since the comparison between the expected and the actual fatty acid levels do not match, which suggests that propionyl-CoA plays a minor role. C17:0 could be biosynthesised through α-oxidation of C18:0, which was investigated in the subsequent studies.

The target substrate for α-oxidation is phytanic acid has been shown to competitively inhibit C17:0 biosynthesis (see the phytol supplementation study). As shown in Supplementary appendix 19 we investigated the diet composition of phytol and phytanic acid, comparing these for each diet in the ruminant-fat dose response study in rats. The dietary combination of phytanic acid and the phytol had a direct inverse correlation with the changes in the plasma C17:0 (as dietary phytanic acid and phytol increases the plasma C17:0 decreases proportionately), which is reinforced by the results in the mouse phytol supplementation study, and explains why the smallest difference between C17:0 in each diet is between diet groups one and two, where there is actually the largest difference in C17:0 levels in plasma. The correlations between circulating C17:0 and the dietary phytol/phytanic acid in the diet are stronger than with the dietary contributions of C17:0 which suggests that C17:0 is not a direct ruminant-fat biomarker but actually a product of biosynthesis regulated by dietary substrate intake.

In the human dairy fat supplementation study the plasma C15:0 level increased by ~10% (p = 0·031) as a result of a dietary 30% increase, this directly concurs with the expected increase according to the ruminant-fat dose response study (see Fig. 1). However, the plasma C17:0 levels did not change across the supplementation study; agrees with previous findings^29^. This can be explained by the levels of phytanic acid and its precursor phytol. The human supplementation included an increase of phytol by ~25% and an increase of phytanic acid by ~100%, which both reduces the biosynthesis of C17:0. This reduction in the biosynthesis along with the increased dietary consumption of C17:0 accounts for no change seen in the plasma.

To further investigate this, we performed analyses in a controlled, high or low dairy fat diet intervention following a baseline diet in healthy volunteers. For the low dairy fat diet, the C15:0 levels decreased as expected and increased for the high dairy fat intervention participants. The percentage increase and decrease of C15:0 in either of the experimental diets agrees with the expected changes, according to the linear relationship seen in figure one further reinforcing the biomarker relationship of C15:0. The changes in the plasma C17:0 can be explained by the variation in the phytol and phytanic acid in the diet. In the experimental diets there was an increase in phytol by 42% and phytanic acid varied by ~100% to ~230% from the baseline diet. The increases in the target substrates of α-oxidation considerably decreases C17:0 biosynthesis via α-oxidation by competitive inhibition, even when there was an increase in ruminant-fat.

Since it is well understood that C18:0 can be the substrate for C17:0 when studied *in vitro*, we conducted a C18:0 infusion study in rats to assess the effect on *in vivo* C17:0 levels. The absolute serum quantities of C17:0 increased significantly by ~70% (p < 0·001) in the rats receiving the C18:0 infusion compared to a control rat group. Further increasing the infusion of C18:0 above 250 nmol/kg/day did not further increase the serum C17:0 levels. This suggests that C18:0 itself does not increase the rate of C18:0 α-oxidation.

We also showed that *Hacl1* plays a substantial role in the biosynthesis of C17:0. Using the *Hacl1* knockout mouse model, there was a 32·5% reduction in plasma C17:0 compared to wildtype mouse controls. There were no other differences in any other fatty acids measured. It is unlikely to expect a complete C17:0 deficiency since there are other salvage pathways that could compensate for a deficiency in *Hacl1*; such as hydroxylation by fatty acid 2-hydroxylase (FA2H) followed by oxidation by 2-hydroxy acid oxidase (HAO2), additionally, dietary C17:0 sources still contribute to circulating levels.

The significance of the different associations of C15:0 and C17:0 with glucose intolerance has been previously reported, however, no consideration for the factors affecting biosynthesis of C17:0 has been taken into account. We compared the baseline fatty acid levels with the change in glucose and insulin measured during an oral glucose tolerance test after a high fat diet in a suitable canine model; the canine model was selected due to the sample volume requirements of the study and it has been previously shown that dogs are a suitably comparable model to human glucose intolerance pathology. There was no correlation between C15:0 at baseline with the change in either glucose or insulin across the high fat diet, however, a strong correlation was seen with C17:0. These results highlight the importance of understanding the origin of these fatty acids and indicate that the biosynthesis (not the dietary source) of C17:0 has a strong relationship with the development of glucose intolerance, either as a marker of healthy lipid metabolism or indeed the fatty acid may have protective properties.

To summarise, it is clear that individual OC-FAs are the result of very different processes and that their relations with disease risk cannot be generalised. From our results we can conclude that C15:0 is a direct, linear biomarker of dietary C15:0 intake. C17:0 has been misidentified as a ruminant fat intake biomarker due to its relationship with dietary phytol, phytanic acid and C18:0. However, the primary relationship between diet and *in vivo* C17:0 levels is highly influenced by biosynthesis. Moreover, the disparity between C15:0 and C17:0 agrees with data collected in epidemiological studies showing that C17:0 has a stronger inverse association with diseases. This highlights the importance for further investigation into the biosynthesis of C17:0 and how these pathways relate to metabolic disease.

## Additional Information

**How to cite this article**: Jenkins, B. J. *et al*. Odd Chain Fatty Acids: New Insights of the Relationship Between the Gut Microbiota, Dietary Intake, Biosynthesis and Glucose Intolerance. *Sci. Rep.* **7**, 44845; doi: 10.1038/srep44845 (2017).

**Publisher's note:** Springer Nature remains neutral with regard to jurisdictional claims in published maps and institutional affiliations.

## Supplementary Material

Supplementary Material

## Figures and Tables

**Table 1:** The pentadecanoic acid (C15:0) and heptadecanoic acid (C17:0) fatty acid levels (Mol%) of the human plasma samples from the start of the intervention (day = 0) to the end of the intervention (day = 56), where the participants received a dairy fat supplementation of 760 kcal/day for 56 days.

	Day = 0 (Mol %)	Day = 56 (Mol %)	Average % change	p-value
(C15:0)	0.097 ± 0.013	0.106 ± 0.020	9.98	0.031*
(C17:0)	0.144 ± 0.018	0.145 ± 0.016	0.16	0.951

The samples were analysed by gas chromatography with mass spectrometry detection. The diet increase of each of the fatty acids due to the intervention is 30% above baseline. Values are given with ± standard deviation. Differences between the start of the study (Day = 0) and the end (Day = 56) were determined by a paired t-test (a measure of the significance/insignificance of an observation in one sample set (Day = 0) that is paired with the same observation in the second sample set (Day = 56) within the same study population); a value of p ≤ 0.05 was considered significant. (n = 26).

**Table 2:** The changes in pentadecanoic acid (C15:0) and heptadecanoic acid (C17:0) levels (Mol%) from the end of the baseline diet to the end of the experimental diet; represented as change in Mol%.

	Low dairy fat (Change in Mol%)	p-value	High dairy fat (Change in Mol%)	p-value
(C15:0)	−0.374	0.029	0.126	0.034
(C17:0)	−0.192	<0.0001	−0.271	<0.0001

The plasma samples were analysed by direct infusion with mass spectrometry detection. A significant change from the end of the baseline diet to the end of the experimental diet is represented by the homoscedastic t-test p-value (p ≤ 0.05 was considered significant). (n = 64).
