# Supplementary material for: Odd Chain Fatty Acids; New Insights of the Relationship Between the Gut Microbiota, Dietary Intake, Biosynthesis and Glucose Intolerance
Source: Sci Rep. 2017 Mar 23;7:44845. doi: 10.1038/srep44845 (PMC5362956; doi:10.1038/srep44845)
Supplement: Supplementary Material [file srep44845-s1.pdf]

SUPPLEMENTARY MATERIAL

Odd Chain Fatty Acids: New Insights of the Relationship Between the Gut Microbiota, Dietary Intake, Biosynthesis and Glucose Intolerance.

Benjamin J. Jenkins; Kevin Seyssel; Sally Chiu; Pin-Ho Pan; Shih-Yi Lin; Elizabeth Stanley; Zsuzsanna Ament; James A. West; Keith Summerhill; Julian L. Griffin; Walter Vetter; Kaija J. Autio; J. Kalervo Hiltunen; Stéphane Hazebrouck; Renata Stepankova; Chun-Jung Chen; Maud Alligier; Martine Laville; Mary Moore; Guillaume Kraft; Alan Cherrington; Sarah King; Ronald M. Krauss; Evelyn de Schryver; Paul P. Van Veldhoven; Martin Ronis; Albert Koulman*.

Supplementary appendix 1: Animal care ethical approval statement for the influence of non-ruminant gut microbiota during a 5% low fat (NB. 0% ruminant fat content) diet on the *in vivo* levels of odd chain fatty acids.
All experiments were performed with permission 91-493 of the French Veterinary Services and in accordance to the European Community rules of animal care.

Supplementary appendix 2: Animal care ethical approval statement for the influence of non-ruminant gut microbiota during a 35% high fat (NB. 13% ruminant fat content) diet on the *in vivo* levels of odd chain fatty acids.
The study was performed in the department of Immunology and Gnotobiology of the Institute of Microbiology, Czech Academy of Science. The institute is authorised by the Central Committee for Animal Welfare to carry out experiments on laboratory animals and the local ethics guidelines are in compliance with Directive 86/608/EEC on the protection of animals used for scientific purposes and recommendation 2007/526/EC of the European Commission.

Supplementary appendix 3: Animal care ethical approval statement for the influence of a ruminant fat (NB. 0% to 11.7% ruminant fat content) dose response study on *in vivo* levels of odd chain fatty acids.
All experimental procedures were ethically approved by the Institutional Animal Care and Use Committee at the University of Arkansas for Medical Science.

Supplementary appendix 4: Animal care ethical approval statement for the influence of a controlled 14 day dietary phytol supplementation study (NB. An additional 0.5% w/w) on the *in vivo* levels of odd chain fatty acids.
All experiments were executed according to accepted criteria for the humane care and experimental use of laboratory animals. All protocols were approved by the Animal Care and Use Committee of the University of Oulu.

Supplementary appendix 5: Ethical approval statement for the human intervention study where the participants received a dairy fat supplementation of 760 kcal/day for 56 days.
All the participants gave signed consent following the explanation of the experimental protocol. The protocol was approved by the ethics committee of Lyon Sud-Est according to the French 'Huriet-Serusclat' law and the Second Declaration of Helsinki (registered study number NCT00905892).

Supplementary appendix 6: Ethical approval statement for the randomly controlled human intervention study where the participants followed a 28 day baseline diet then randomly assigned to one of two isocaloric experimental diets; high dairy fat or low dairy fat, for a further 28 days.
Written informed consent was provided and the protocol was reviewed and approved by the institutional review boards of Children's Hospital & Research Center at Oakland (dba University of California San Francisco Benioff Children's Hospital Oakland) and the University of California, San Francisco (registered study number NCT01404897).

Supplementary appendix 7: Animal care ethical approval statement for the influence of a controlled two dose 35 day intraperitoneal infusion of stearic acid on the *in vivo* levels of odd chain fatty acids.

The animal study was approved by the Animal Care and Use Committee of Taichung Veterans General Hospital, Taiwan.

Supplementary appendix 8: Animal care ethical approval statement for the influence of a controlled 2-hydroxyacyl-CoA lyase 1 gene knockout mouse model on the in vivo levels of odd chain fatty acids.
All animal experiments were approved by the Institutional Animal Ethical Committee of KU Leuven.

Supplementary appendix 9: Animal care ethical approval statement for the comparison of baseline odd chain fatty acid levels on the prognosis of diet induced glucose intolerance.
The protocol was approved by the Vanderbilt University Institutional Animal Care and Use Committee, and the animals were housed and cared for according to Association for Assessment and Accreditation of Laboratory Animal Care guidelines.

Supplementary appendix 10: Sample preparation.
To obtain serum, blood was collected and allowed to clot by leaving undisturbed at room temperature for ~30 minutes, then centrifuge at ~1500 g for 10 minutes. Remove the supernatant (serum) and store at sub -20ºC until analysed. To obtain plasma, blood was collected in commercially available anticoagulant-treated tubes (EDTA, citrate or heparin) and centrifuged at ~1500 g for 10 minutes. Remove the supernatant (plasma) and store at sub -20ºC until analysed.

Supplementary appendix 11: Study design for the influence of non-ruminant gut microbiota during a 5% low fat (NB. 0% ruminant fat content) diet on the *in vivo* levels of odd chain fatty acids.
Two groups of BALB/cByJ mice (n=8 per group) were used[1]; conventional, specified and opportunistic pathogen free mice were purchased from Charles River Laboratories (Charles River Laboratories, L'Arbresle, France). Germ-free mice were bred in the ANAXEM platform germ-free animal facilities (ANAXEM platform, INRA, Jouy-en-Josas, France). All animals were housed in sterile Trexler-type isolators (Getinge-La Calhène, Vendôme, France). Autoclaved tap water and sterile pelleted low-fat diet (R03, SAFE, Augy, France) were provided *ad libitum*. Fatty acid composition of the diet (Mol %): C15:0 = 0.035, C17:0 = 0.089. The absence of gut microbiota in the germ-free mice was monitored by aerobic and anaerobic culture examinations of faecal preparations. Blood was collected when the mice were 12 weeks old and processed into serum, this was then stored at sub -20ºC until analysed by gas chromatography with mass spectrometry detection.

Supplementary appendix 12: Study design for the influence of non-ruminant gut microbiota during a 35% high fat (NB. 13% ruminant fat content) diet on the *in vivo* levels of odd chain fatty acids.
For the investigation of the effects of high-fat feeding, two groups of C57/BL6 mice (n=6-7 per group) were used; conventional mice with complete normal intestinal microbiota and germ-free mice. Intestinal colonization of the conventional mice was achieved through administration of specific pathogen free bacterial cocktail (Central Institute for Laboratory Animal Breeding, Hanover) containing both aerobic microorganisms (*Bacillus species, Lactobacillus lactis, Acinetobacter species* and *Staphylococcus sciuri*) and anaerobic microorganisms (*Lactobacillus delbrukii sp., Bulgaricus, L. fermentum, L. Catenaforme, Bacteroides distasonis, Bacteroides thetaiotaomicron, Peptostreptococcus micros, P. asaccharolyticus*). The germ-free mice were bred under sterile conditions for the past four generations with faecal samples evaluated weekly for the absence of aerobic and anaerobic bacteria, mould and yeast. The conventional mice were evaluated for the presence of bacteria after 2 days, 7 days and then every week following the colonisation. Faecal samples were cultivated on peptone bouillon, Sabouraud bouillon, Schadller bouillon (Difco, Detroit, MI) and then on blood agar, Sabourad agar, Schadller agar in anaerobic atmosphere (gas Anaerogen 2.5lt:DIOXO) to detect any presence of anaerobic bacteria. Smears were stained with Gram stain and fluorescence dyes[2], which stained Gram-positive bacteria yellow and Gram-negative bacteria green. After weaning, both groups of mice received sterilised water and pelleted 35% high-fat feed (C1090-60, Altromin, Lage, Germany) with 13% milk-fat and 22% lard-fat ad libitum. Fatty acid composition of the diet (Mol %): C15:0 = 0.953, C17:0 = 0.978. Diet and bedding material were sterilised with gamma radiation (25 kGy, 30 min; Bioster, Czech Republic). Blood was collected after 75 days and processed into plasma, this was then stored at sub -20ºC until analysed by gas chromatography with mass spectrometry detection.

Supplementary Table appendix 13: The fat source and pentadecanoic acid (C15:0) and heptadecanoic acid (C17:0) composition of the five experimental diets used in the dose response study in rats where five groups were subjected to individual isocaloric high-fat diets with increasing ruminant fat content from 0% to 11.7%.

	Fat source			Fatty acid composition	
Diet	Corn oil	MCT oil	Beef tallow	C15:0 (Mol %)	C17:0 (Mol %)
1	70%	0%	0%	0.007	0.073
2	50%	16.4%	3.6%	0.050	0.090
3	35%	28.7%	6.3%	0.084	0.120
4	20%	41%	9%	0.118	0.150
5	5%	53.3%	11.7%	0.152	0.179

N.B. The C17:0 composition was higher than then C15:0 due to the beef tallow used in the experimental diet.

Supplementary appendix 14: The eligibility and exclusion criteria for the randomly controlled human intervention study where the participants followed a 28 day baseline diet then randomly assigned to one of two isocaloric experimental diets; high dairy fat or low dairy fat, for a further 28 days.

Eligibility criteria included: age ≥ 18 years, body mass index 25-40 kg / m2, homeostatic model assessment-insulin resistance (glucose X insulin / 22.5) ≥ 2.5, fasting blood glucose < 7.0 mmol / L, plasma TGs < 5.65 mmol / L and total & LDL cholesterol ≤ 95th percentile for age and sex. Exclusion criteria include: currently smoking or history of diabetes, cardiovascular disease or other chronic disease, additionally, any use of drugs known to affect glucose or lipid metabolism, blood thinning agents, dietary supplements or hormones.

Supplementary Table appendix 15: The composition of the baseline and the two experimental diets used in the 28 day intervention study with a normalising 28 day baseline diet phase. Values are shown as percentage of total energy intake (3000 kcal menu). The participants were provided with lunch and evening meals (Lifespring Home Nutrition, California, USA) based on a seven day rotational menu. The participants were required to purchase and prepare their morning meal and snacks according to the menus provided. High-fat and non-fat dairy products were used to vary dairy fat intake across the two experimental diets. All diets met the recommended dietary allowances for vitamin and mineral intake.

		Experimental diets	
	Baseline diet (%)	High dairy fat (%)	Low dairy fat (%)
Protein	15	30	30
Carbohydrates	55	35	35
Total fat	30	35	35
Dairy fat	9	14	8

Supplementary appendix 16: The materials and fatty acid methyl ester sample preparation procedure followed by gas chromatography with mass spectrometry detection.

Chemicals were obtained from Sigma-Aldrich (Sigma-Aldrich Company Ltd, Dorset, UK). All solvents were of HPLC grade.

Samples were extracted using the chloroform: methanol: water extraction, adapted from the previously described method by Folch et al (Folch et al., 1957). Briefly, chloroform: methanol solution (2: 1, 1 mL) was added to 100 µL of plasma or serum. The samples were then vortexed and sonicated for 15 minutes. Water (400 µL) was added to each sample, followed by further vortexing then sonication (15 min), and additional vortexing to ensure complete recovery. Samples were centrifuged (20,238 g, 5 min) and the resulting aqueous and organic layers were separated and dried under a gentle stream of nitrogen.

For the analysis of total fatty acids, the samples were derivatised. To the dried organic extracts; boron trifluoride in methanol (14%, 125 µL), chloroform: methanol (1:1, 100 µL) and internal standard tridecanoic acid-d25 in chloroform (100 µL, 200 µMol) were added. The mixture was thoroughly vortexed, sonicated (30 min) and vortexed a further time to ensure there was no undissolved material. Samples were heated to 80°C for 90 minutes to commence the derivatisation process.

After the samples had cooled, water (300 µL) and hexane (600 µL) were added. The samples were vortexed and the organic layer separated into glass vials, blown down to dryness under nitrogen and finally reconstituted in 200 µL of hexane, ready for gas chromatography with mass spectrometry detection (GC-MS) analysis.

Gas chromatography separation was achieved using a 6890N / 5973 Agilent GC-MS system (Agilent Technologies, California, USA) with a HP-88, 30 meter capillary column, 0.25 mm internal diameter and a 0.2 µm film thickness (Agilent 112-8837). Inlet temperature of 250oC. A total oven gradient over 26 minutes from 120oC to 210oC (initial temperature 120oC hold of 1 minute, temperature increase of 10oC per minute to 170oC followed by a hold of 6 minutes, then temperature increase of 3oC per minute to 210oC followed by a hold of 1 minute) with a 5:1 split. Full scan mass spectrometry detection starting after a delay of 2 minutes (mass range from 60-400 Da, transfer line temperature of 280ºC, MS-source temperature of 230oC, MS-quadrupole temperature of 150oC). Peaks were integrated using GC/MSD ChemStation (Agilent Technologies, California, USA) and signal intensities were normalised to the internal standard.

Supplementary appendix 17: The materials, lipid extraction, and fatty acid analysis by direct infusion with mass spectrometry detection.

Lipid analytes were extracted from plasma samples by a modified version of the methyl-tert-butyl ether (MtBE) extraction method published by Matyash and co-workers[3]. Briefly, 100 µL of water was added to 15 µL of plasma / serum and thoroughly vortexed. Then the internal standard was added; heptadecanoic acid-d33 (150 µL, 8 µMol), followed by 750 µL of MtBE, the mixture was then thoroughly vortex to ensure maximum analyte recovery. Then 200 µL of water was added to produce separate organic and aqueous fractions. 25 µL of the MtBE fraction (upper layer) was transferred to a glass coated 96-well plate and reconstitute with 90 µL of 2:1 isopropanol to methanol mix, including 7.5 mMol of ammonium acetate.

Direct infusion of the samples was carried out using a TriVersa NanoMate (Advion, Ithaca, USA), briefly; 10 µL of the prepared sample was sprayed for 2 minutes, with a back pressure of 0.4 psi, voltage of -1.4 kV in negative mode. Full mass spectrometry detection (Exactive Orbitrap, Waltham, USA) was started after a 20 second delay to allow enough time for the spray to stabilise, data were acquired for 2 minutes per sample. The mass spectrometer used the following parameters: mass range of 185-500 Da, transfer capillary temperature of 350oC, resolution of 100,000 @ 1 Hz, with Collision-induced dissociation energy of 100 eV.

Peaks were integrated using an integration script in R (R Development Core Team (2008). R: A language and environment for statistical computing. R Foundation for Statistical Computing, Vienna, Austria. ISBN 3-900051-07-0, URL http://www.R-project.org.), where the total signal area of the fatty acid exact mass was calculated over the two minute infusion.

The R integration script is; setwd("#1") #1 where your mzXML files are stored… source("#2") #2 directory where the hrms_POS.R script is stored… library(xcms)… library(data.table) … files = list.files(".", pattern=".mzXML") … system.time (for (i in 1:length(files)) {main(files[i], rtwin=c(0,120), mzwin=c(185,500))}) … results <- signals_deviations().

The fatty acids with their associated masses (m/z) integrated with this R script are: C14:0 (m/z = 227.2017), C14:1 (m/z = 225.1860), C15:0 (m/z = 241.2173), C16:0 (m/z = 255.2330), C16:1 (m/z = 253.2173), C17:0 (m/z = 269.2486), C18:0 (m/z = 283.2642), C18:1 (m/z = 281.2486), C18:2 (m/z = 279.2330), C18:3 (m/z = 277.2173), C20:3 (m/z = 305.2486). Signal intensities were normalized to the internal standard heptadecanoic acid-d33 (m/z = 302.4557).

Supplementary Figure appendix 18: Results for the two controlled gut microbiota investigations; 5% low fat (NB. 0% ruminant fat content) diet and a 35% high fat (NB. 13% ruminant fat content) diet on the *in vivo* levels of odd chain fatty acids.

The low-fat diet consisted of 5.1% fat, 21.4% protein and 55.7% carbohydrates and the high-fat diet consisted of 35% fat, 21.4% protein and 34.4% carbohydrates. The differences in the OC-FA levels between conventional and germ-free animals were not significant, this observation was independent of the diet. As expected the levels of OC-FAs were greater in the high-fat diet animals compared to the low-fat diet animals.

Box and whiskers plot showing the influence of intestinal microbiota on plasma odd chain fatty acids in mice, values are percentage of total fatty acids measured by gas chromatography with mass spectrometry detection. Box represent mean ± standard deviation, whiskers represent the minimum and the maximum values. Pentadecanoic acid (C15:0) and heptadecanoic acid (C17:0), low-fat diet (LF) and high-fat diet (HF), conventional mice (CV) and germ-free mice (GF). (n=6-8 per group).

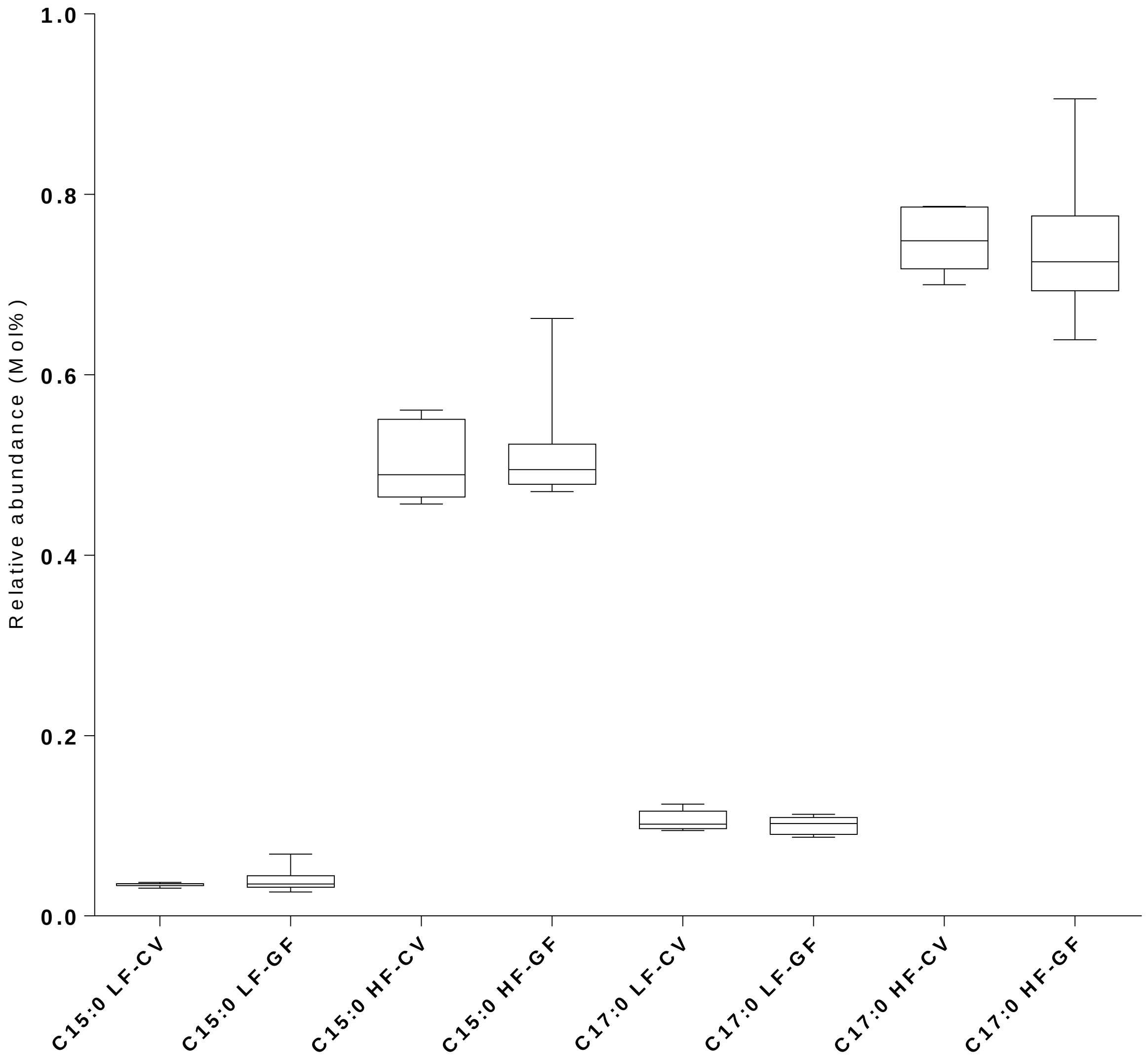

Supplementary Figure appendix 19: The composition of the sum of phytol and phytanic acid in each isocaloric high fat diet used in the ruminant fat (0% to 11.7%) dose response study. Values shown as milligrams per 100 grams of lipid source analysed.

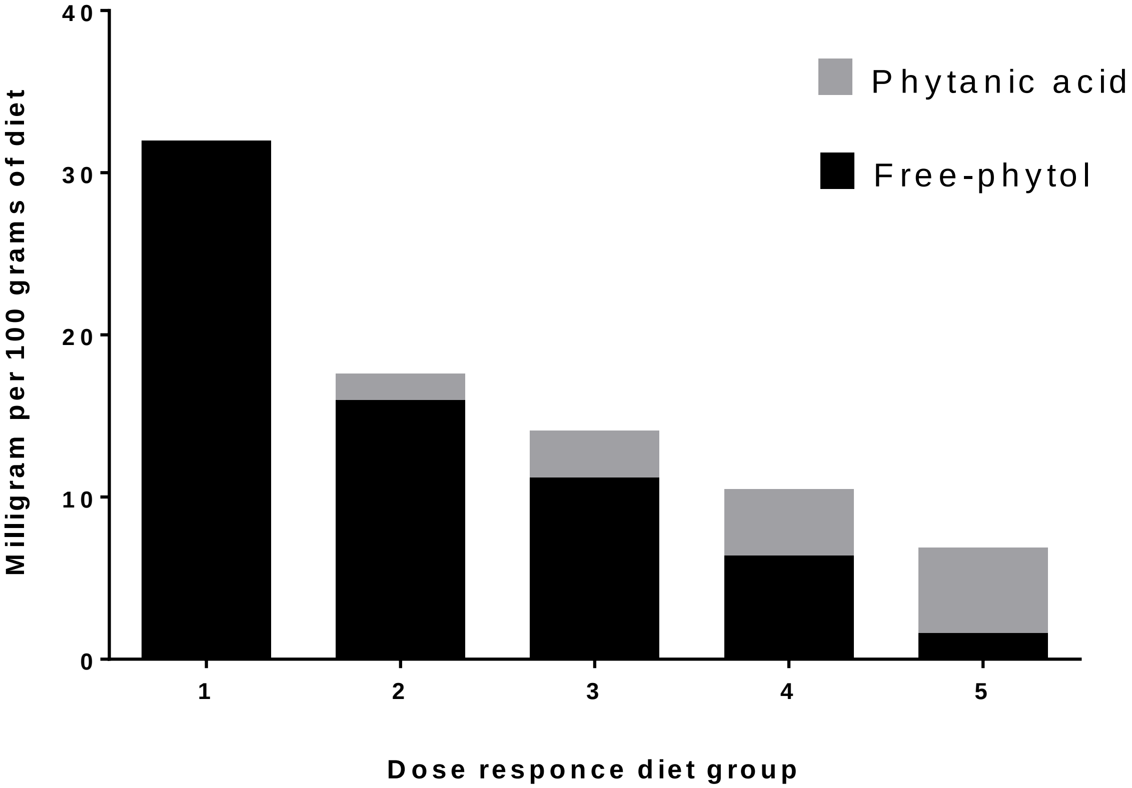

Supplementary Table appendix 20: The levels (Mol%) of pentadecanoic acid (C15:0), heptadecanoic acid (C17:0), phytol and phytanic acid for each fat source consumed during the human intervention study where the participants received a dairy fat supplementation of 760 kcal/day for 56 days. Values are shown as milligrams per 100 grams of the fat source. The actual number of milligrams consumed per day is shown in the 'intervention' column.

	Cheese (mg / 100 g)	Butter (mg / 100 g)	Almonds (mg / 100 g)	Intervention (mg)
(C15:0)	350	880	8	529
(C17:0)	200	330	30	278
Phytol	0	2.25	2.3	1.37
Phytanic acid	54	177	0	89.4

Supplementary Table appendix 21: The dietary intervention adherence data for the human intervention study where the participants received a dairy fat supplementation of 760 kcal/day for 56 days. The dietary assessment of the human participants throughout the time course (day=0 to day=56) of the intervention showing that the dairy intake has significantly increased due to the supplementation, 234.1 to 308.1 grams per day. This table also confirms that the participant habitual diet remained compositionally unchanged throughout the intervention. Values are given with ± standard deviation. (n=26).

	Day 0	Day 56
Energy intake (kcal / day)	2236 ± 71	3040 ± 76
Protein intake (g / day)	92.1 ± 3.1	127.0 ± 3.0
Carbohydrate intake (g / day)	254.3 ± 8.4	270.6 ± 10.9
Lipid intake (g / day)	93.6 ± 4.7	160.3 ± 5.1
Saturated fatty acid (g / day)	35.7 ± 2.1	62.9 ± 1.8
Mono-unsaturated fatty acid (g / day)	28.5 ± 1.7	52.1 ± 1.9
Poly-unsaturated fatty acid (g / day)	11.2 ± 0.8	19.6 ± 1.1

Dairy intake (g / day)	234.1 ± 23.7	308.1 ± 23.7

Supplementary Table appendix 22: The daily average intake (mg/day) of pentadecanoic acid (C15:0), heptadecanoic acid (C17:0), phytol, and phytanic acid for the baseline diet and each of the experimental diets used in the randomly controlled human intervention study; where the participants followed a 28 day baseline diet then randomly assigned to one of two isocaloric experimental diets; high dairy fat or low dairy fat, for a further 28 days.

		Experimental diets	
	**Baseline/control diet (mg)**	**Low dairy fat (mg)**	**High dairy fat (mg)**
C15:0	218.1	178.0	330.7
C17:0	223.3	173.5	274.6
Phytol	1.2	1.7	1.7
Phytanic acid	30.2	30.7	68.3

N.B. The higher increase of C15:0 than C17:0 in the ‘high dairy fat’ diet is due to the higher fatty acid composition in dairy-fat, roughly 2:1 C15:0 to C17:0 respectively.

Supplementary Figure appendix 23: Healthy adult dogs (n=5) were fed a high fat diet (4-8 weeks) where they had their glucose tolerance assessed by an oral glucose tolerance test at the start (x) and end (■) of the intervention. The points represent the average insulin concentration (pmol/L) for each time point (minutes).

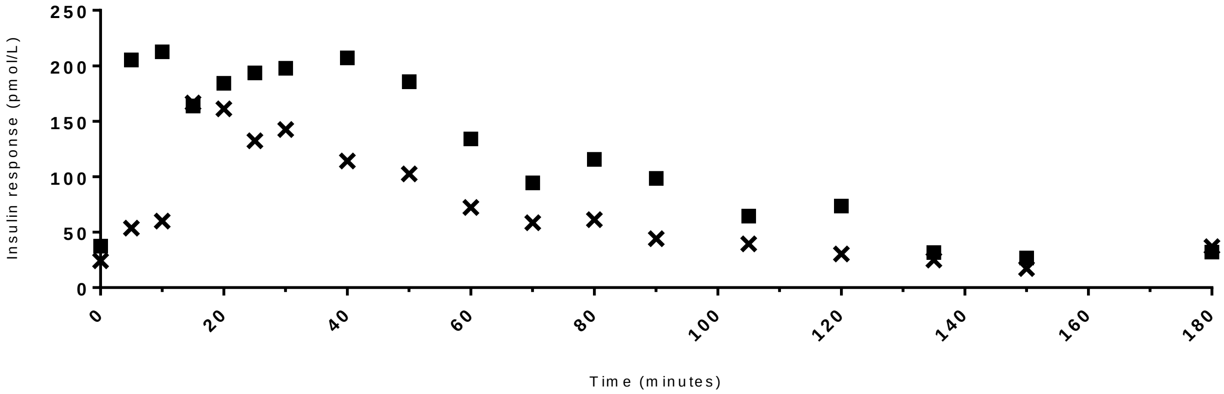

Supplementary Figure appendix 24: Healthy adult dogs (n=5) were fed a high fat diet (4-8 weeks) where they had their glucose tolerance assessed by an oral glucose tolerance test at the start (x) and end (■) of the intervention. The points represent the average glucose concentration (mmol/L) for each time point (minutes).

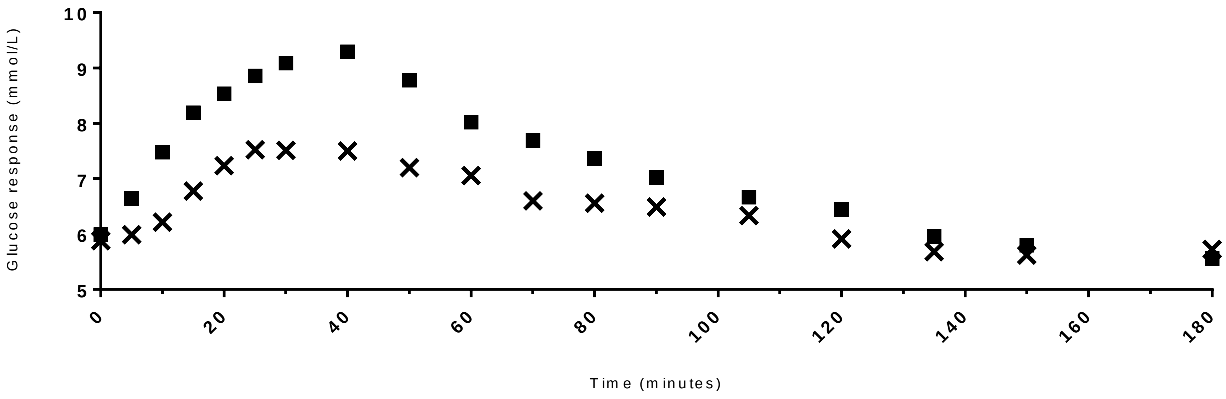

References

1. Morin S, Bernard H, Przybylski-Nicaise L, Corthier G, Rabot S, Wal J-M, et al. Allergenic and immunogenic potential of cow's milk β-lactoglobulin and caseins evidenced without adjuvant in germ-free mice. Mol Nutr Food Res. 2011 Nov 1;55(11):1700–7.
2. Stepankova R, Tonar Z, Bartova J, Nedorost L, Rossman P, Poledne R, et al. Absence of microbiota (germ-free conditions) accelerates the atherosclerosis in ApoE-deficient mice fed standard low cholesterol diet. J Atheroscler Thromb. 2010;17(8):796–804.
3. Matyash V, Liebisch G, Kurzchalia TV, Shevchenko A, Schwudke D. Lipid extraction by methyl-tert-butyl ether for high-throughput lipidomics. J Lipid Res. 2008 May 1;49(5):1137–46.
